# Attraction of *Culex pipiens* to House Sparrows Is Influenced by Host Age but Not Uropygial Gland Secretions

**DOI:** 10.3390/insects9040127

**Published:** 2018-09-25

**Authors:** Mary C. Garvin, Amy Austin, Kevin Boyer, Madeleine Gefke, Celestina Wright, Yemko Pryor, Anah Soble, Rebecca J. Whelan

**Affiliations:** 1 Department of Biology, Oberlin College, Oberlin, OH 44074, USA; amy.austin181@gmail.com (A.A.); kboyer@oberlin.edu (K.B.); mgefke@oberlin.edu (M.G.); cwright2@oberlin.edu (C.W.); ypryor@oberlin.edu (Y.P.); Anah.Soble@oberlin.edu (A.S.); 2 Department of Chemistry and Biochemistry, Oberlin College, Oberlin OH 44074, USA; rwhelan1@nd.edu; 3 Department of Chemistry and Biochemistry, University of Notre Dame, Notre Dame, IN 46556, USA

**Keywords:** mosquitoes, behavior, chemical biology, ecology

## Abstract

*Culex pipiens* serves as the endemic vector of West Nile virus (WNV) in eastern North America, where house sparrows (HOSP, *Passer domesticus*) serve as a reservoir host. We tested the hypotheses that: (1) Attraction of *Cx. pipiens* to HOSP is influenced by bird age and (2) that age-specific variation in chemical profiles of bird uropygial gland secretions informs this choice. We conducted mosquito choice trials in an olfactometer and found that *Cx. pipiens* were more often attracted to adult sparrows over nestlings, however, they demonstrated no preference for adults over fledglings. Using gas chromatography-mass spectrometry we observed age-specific differences in the semi-volatile chemical profiles of house sparrow uropygial gland secretions. Contrary to our hypothesis, we found no significant difference in mosquito feeding preference between the secretions of adults and those of either nestlings or fledglings. We suggest that other chemical cues influence the feeding preference of *Cx. pipiens*, either independently of uropygial gland secretions, or synergistically with them.

## 1. Introduction

In the eastern United States, West Nile virus (WNV) is maintained in bird reservoir host populations by *Cx. pipiens* [1], which survives high viral titers [2], is abundant [3], and feeds readily on birds [2]. The factors that influence the interaction between the reservoir hosts and the mosquito vectors are therefore key to the maintenance of the cycle. Nestling hosts have been hypothesized to play greater roles than adults in arboviral transmission in nature [4,5] because they have relatively less plumage and mobility [6], as well as a weaker immune response [7]. Other studies report that Culex mosquitoes show no age-based preference [8] and concluded that nestling birds were not important amplification hosts in the WNV cycle in their region [9]. Thus the role of host age in the WNV cycle remains under debate.

Scott et al. [10] studied the attraction of *Cx. quinquefasciatus* to house sparrows (HOSP, *Passer domesticus*) and reported a preference for adults over nestlings. While the basis for this preference was not known, they speculated that olfaction was likely important. Mosquitoes locate blood meals in part through carbon dioxide, but as a by-product of respiration, it alone cannot explain why mosquitoes demonstrate species-specific host preferences [10,11,12]. Thus host choice is likely influenced by more species-specific chemical profiles [11,13]. One source of chemical cues that mosquitoes may use is the uropygial gland. Located at the base of the tail, the gland is the source of a diverse mixture of organic compounds [14] that birds spread over their feathers during preening. In addition to waterproofing and conditioning the feathers [15], the secretions may also function in intraspecific and interspecific communication (see reviews [16,17]). The role of the gland in *Cx.* spp. feeding behavior remains unclear. *Culex pipiens* has demonstrated preference for light traps baited with uropygial secretions of crows [18]. In contrast, Bernier et al. [19] found that *Culex* spp. were not attracted to components of chicken uropygial secretions; however, they suggested that mosquitoes might cue into compounds that result from the degradation of secretions by microbes. Likewise, Allan et al. [20] reported that *Culex* spp. were not attracted to either extracted feather volatiles or uropygial diols in isolation, but were attracted to whole feathers, suggesting a synergistic effect.

In the current study, we used HOSP, an avian reservoir host of WNV in eastern North America, to evaluate age-specific preferences of *Cx. pipiens* and the influence of uropygial gland secretions on that preference. Based on the work of Scott et al. [10], we predicted that *Cx. pipiens* will be more often attracted to adult house sparrows than to juveniles (either nestling or fledgling). We then hypothesized that age-based variation in semi-volatile components of HOSP uropygial gland secretions would influence the mosquito preference. To examine this hypothesis, we tested the following predictions: (1) Semi-volatile profile of secretions will vary with age and (2) *Cx. pipiens* will be more attracted to the secretions of the preferred age group.

## 2. Materials and Methods

### 2.1. Chemical Analysis of Uropygial Gland Secretions

Chemical standards, GC-MS sample analysis, and secretion component identification methods used in this study follow those described in Garvin et al. [21]. Briefly, uropygial secretions were weighed by difference and dissolved to 20 mg/mL using dichloromethane as the diluent. Each sample also contained 0.005% (*v*/*v*) benzyl alcohol to serve as an internal standard. Dissolved samples were heated to fully dissolve and centrifuged to remove residual solids. 1 µL of supernatant was injected into a Trace GC gas chromatograph coupled with a Polaris Q mass analyzer. Chromatography was accomplished using a non-polar column and a thermal ramp optimized for resolution of the compounds found in the secretions. Mass spectrometry standards were prepared by methyl esterification of carboxylic acids ranging from 16 to 20 carbons in length. Standards were analyzed using the same GC-MS conditions as the uropygial secretion samples.

#### 2.1.1. Bird Handling

Adult and fledgling house sparrows were caught using both mist nets and traps at a dairy farm approximately one mile south of Oberlin and on the Oberlin College campus. Sex was determined via brood patch, plumage, or identification of a sex-specific CHD gene using polymerase chain reaction (PCR) and gel electrophoresis [21]. For the latter protocol, approximately 0.05 mL of blood was collected via brachial venipucture into a heparinized microhematocrit tube and one drop was placed in 300 μL of cell lysis solution. After collection of uropygial gland secretions into glass capillary tubes, as described in Garvin et al. [22], birds were measured, weighed, aged according to Pyle [23], and released at the site of capture. Uropygial gland secretions were immediately placed in chromatography vials that were capped and kept in the freezer until analysis. Nestlings were collected from nest boxes on the Oberlin College campus and private residences.

Protocols involving live birds were approved by Oberlin College IACUC # S09RBMG-5 and # S15RMG-2.

#### 2.1.2. Statistics

To test for an effect of age on HOSP secretions, principal components analysis (PCA) was used to reduce the standardized integrated peak areas of the compounds to principal components (PCs), followed by a multivariate analysis of variance (MANOVA) on the PCs. All statistical analyses were performed using PASW Statistics 18 (2009). *p*-values of ≤0.05 were considered significant.

### 2.2. Bioassays

Choice trials were conducted in a dual port olfactometer modified from Posey et al. [24] as described in Garvin et al. [21]. Choice trials were conducted with *Cx. pipiens* from a colony established in 2009 from the Ohio State University, Department of Entomology colony. Larvae were reared in 1000 mL deionized water in 2000 mL glass bowls and fed beef liver powder (Now Foods, Bloomingdale, IL, USA). Adults were reared on 10% sucrose and water at 24 °C, 70–80% humidity, and a 14/10 h (L/D) photoperiod. Eggs were collected in oviposition bowls with grass clippings 5 days after blood meals. Assays were conducted with female mosquitoes between 7 and 21 days of age deprived from access to sugar meals for 24 h. Thirty female mosquitoes were aspirated into the flight chamber and allowed to acclimate for 15 min prior to the start of the trial. Birds or secretions were placed in the two stimulus ports; pairings are described in Table 1.

Each test comprised multiple trials in which 30 female mosquitoes were placed in the flight chamber and allowed to choose either the right or left port for 45 min, and the number of mosquitoes in each port was counted. No birds or mosquitoes were used in more than one trial. To avoid residual odors from the previous trial, the entire olfactometer was cleaned between trials with a solution of 70% ethanol in water and allowed to dry for 20 min. In addition, for within-treatment trials, stimuli were alternated between the left and right port so that no consecutive trials were conducted with stimuli in the same side.

Birds for live bioassays were captured in walk-in traps on Oberlin College campus on the day of assay, and then released at the site of capture. For uropygial gland secretion-only assays, we used secretions collected from fledgling and adult HOSPs during June and July 2012 and stored in GC-MS vials at −80 °C. Secretions were collected and placed inside the stimulus ports in sterile petri dishes as described in Garvin et al. [21], then a plume of carbon dioxide was released into the incurrent air entering the stimulus ports to activate mosquito feeding.

Choices of mosquitoes within a choice trial were not independent of one another, therefore, we used a generalized linear mixed model (SPSS) with trial as a random factor and individual mosquitoes as subjects within trials. We modeled a binary probability distribution with a logit link function in which the port (right or left) that a mosquito moved into during the trial was the response variable, and the side that the adult sparrow was on was the fixed effect predicting mosquito choice. *p*-values of ≤0.05 were considered significant.

## 3. Results

### 3.1. GC-MS Analysis Comparing Uropygial Secretion Composition of Nestling, Fledgling, and Adult House Sparrows

**Figure 1:**
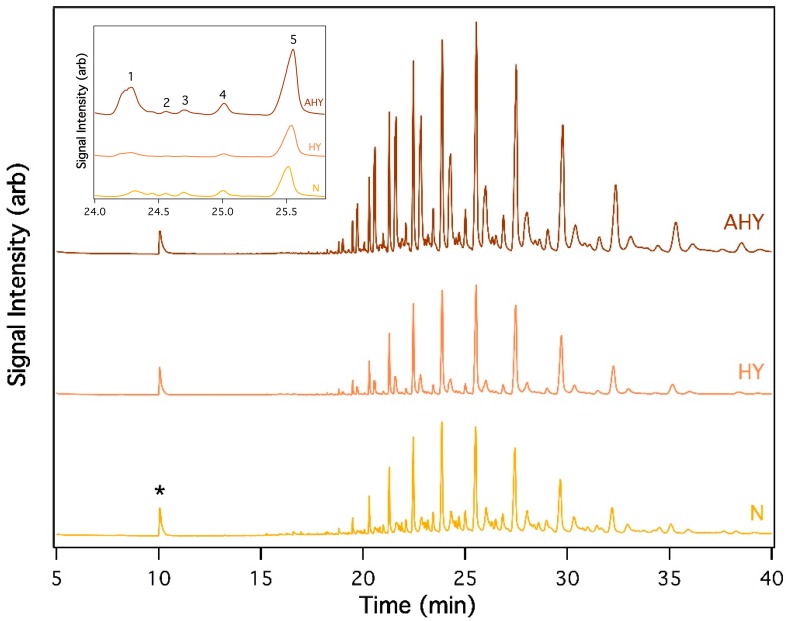
Representative gas chromatograms from uropygial secretions of nestling (N; bottom), hatch year (HY; middle), and adult (AHY; top) house sparrow. Levels of heavy esters (C31–C37) were lower in nestlings (N) than HY (*p* < 0.001) or AHY birds (*p* = 0.02) and HY birds had lower levels than AHY (*p* = 0.013). Lighter esters (C28–C31) were lower in nestlings than HY (*p* = 0.03) and AHY (*p* < 0.001) birds and HY had lower levels than AHY birds (*p* = 0.05). Inset shows an expanded time segment to illustrate the repeated patterns seen in these chromatograms. Peaks 1–5 result from structural isomers with different extents of branching. The asterisk designates the internal standard, benzyl alcohol.

GC-MS analysis indicated that HOSP uropygial secretions contain complex mixtures of semivolatile esters. We classified these esters according to total chain length by examining the mass spectrum for each chromatographic feature, noting that esters consistently produce a parent ion at M + 1. Examination of the total ion chromatograms revealed that several chromatographic features shared the same *m*/*z* value, indicative of different branching patterns giving rise to varied retention times. Since fragmentation in the mass analyzer varies with molecular branching, there was no single characteristic mass spectrum for esters. The esters in the HOSP secretions were found to follow a repeating pattern (Figure 1, inset) that corresponds to five different structural isomers of the same molecular weight. As of the regularity of this pattern, we hypothesize that within each set of esters of the same total molecular weight, each peak possesses the same branching pattern as the corresponding ester in the other sets.

To further characterize the semivolatile esters, we performed methyl esterification reactions on representative secretion samples. GC-MS analysis of the resulting methyl esters enabled the identification of the chain length of the carboxylic acids and alcohols comprising the esters (Table 2).

We used selected ion monitoring and published fragmentation values [25,26] to determine the basic branching pattern of the methyl esterified carboxylic acids. The values used to characterize branching were: *m*/*z* = 101 for 3-methyl branching, *m*/*z* = 88 for 2-methyl branching, *m*/*z* = 87 for 4-methyl branching, and *m*/*z* = 143 for straight chain methyl esters. We were unable to identify branching in the alcohol portions.

Secretions from house sparrows contained abundant and complex semi-volatile compounds with retention times between 18 and 42 min, as shown in Figure 1. The same compounds were present in secretions from birds of different ages, but notably, the relative abundance of esters varied with age (Figure 1).

Age had a significant effect on PC1 (F = 17.332, *p* < 0.001), which represented a set of heavy esters (C31–C37). Nestlings had lower levels of these compounds then HY birds (*p* < 0.001) or AHY birds (*p* = 0.02). HY birds also had lower levels than AHY birds (*p* = 0.013). Age also had a significant effect on PC3 (F = 11.731, *p* < 0.001), which represented a set of lighter esters (C28–C31) with complex branching patterns. Nestlings also had lower levels of these compounds than HY (*p* = 0.03) and AHY (*p* < 0.001) birds. Levels in HY birds were also lower than AHY birds (*p* = 0.05). We found no differences based on sex.

### 3.2. Bioassays

During behavioral assays in which *Cx. pipiens* were provided a choice between live adult house sparrows and nestlings, the stimulus port containing the adults significantly predicted mosquito choice; most mosquitoes chose the port containing the adult (Table 1). However, during trials with secretions only from adults and nestlings, the stimulus port containing adult secretions did not significantly predict mosquito choice.

In assays during which mosquitoes were presented with live adults and live fledglings, the stimulus port containing the adult did not predict mosquito choice; we found no difference in mosquito choice between the two ports. Likewise, no difference in mosquito choice was observed between adult sparrow secretions and those of fledglings.

## 4. Discussion

Consistent with Scott et al.’s study of HOSP and *Cx. quinquefasciatus*, we found that *Cx. pipiens* are more often attracted to live adult HOSP over nestlings. Moreover, we found no preference for adults over fledgling birds, which is somewhat consistent with Scott’s findings that preference for adults decreases with nestling age. From a broader perspective of disease transmission, we acknowledge that although preference contributes to the relative role of a particular reservoir host age group in the arboviral cycles, it is but one aspect of the interactions. Burkett-Cadena [8] underscore that in nature, a number of factors contribute to a reservoir’s relative role, including abundance, immune status, and behavioral and plumage defenses. Still, the preference for adult HOSP over nestlings provided us with the opportunity to investigate the chemical olfactory cues that Scott et al. suggested might be facilitating the observed preference for adults.

We found support for our prediction that the semivolatile components of the uropygial gland secretions would vary with age. Both light and heavy esters were significantly less abundant in nestlings than in either fledglings or adults. We note that the most striking aspect of the house sparrow uropygial secretions was the regular and repeating pattern of five peaks, corresponding to five ester isomers of the same carbon chain length with different branching patterns. As we were unable to completely characterize the exact branching patterns that differentiate the individual peaks, identification of esters in this study was not possible. Still, it is notable that branching can affect the physical properties of the various esters [27] and their chemical function [28]. In each of the sets of five isomers, the most prominent peak is the last, that which presumably has the least amount of branching. Through principal component analysis, we were able to elucidate trends in the production of the esters. We found that the ester represented by the first and fifth prominent peaks also tended to have high variation among age groups. This high level of variability between the secretions of different age groups might suggest that these prominent esters are functionally important.

While this finding was consistent with our prediction and provided support for our hypothesis, during bioassays, we found no evidence that the isolated semi-volatile components of the secretions influenced the observed preference of *Cx. pipiens* for adult HOSPs over nestlings. Despite our negative results, the role of these esters in host-seeking may be worthy of further study in light of the suggestions of Bernier et al. and Allen et al., that microbial degradation of the secretions, or synergistic interactions with other chemical cues emitted by house sparrows could contribute to mosquito feeding choice. Moreover, while gravid female mosquitoes seeking oviposition sites are reported to be both attracted to and repelled by esters [29,30], to our knowledge the role of esters in female blood-seeking behavior has not been studied.

The quantitative difference that we report in the abundance of esters between house sparrow nestlings and older birds may represent a life history trade-off in energy allocation. Among house sparrows, Moreno-Rueda [31] found a positive correlation between uropygial gland size and feather condition, providing evidence for the gland’s importance in feather maintenance. As nestlings do not yet need to allocate energy towards feather condition and protection, resources may instead be diverted towards growth and development.

## 5. Conclusions

In conclusion, we found no support for our hypothesis that uropygial gland secretions influenced *Cx. pipiens* preference for adult house sparrows. Despite the significant difference between the semivolatile chemical profiles of both nestlings and fledglings with adult birds, we found no evidence that *Cx. pipiens’* preference for adult HOSPs over nestlings is influenced by the semivolatile components of HOSP uropygial secretions. We suggest that the age-based attraction of *Cx. pipiens* to HOSP depends on other compounds that act independently of, or synergistically with, the semivolatile components of uropygial gland secretions.

## Figures and Tables

**Table 1:** Comparison of adult house sparrows versus nestlings or fledglings in both live-bird and secretion-only bioassays.

Trial Type	Assay Type	Number of Trials	Number of Mosquitoes Choosing		Mean % (s.d.) Choosing	*F*-Value (d.f.)	*p*-Value
			Adult HOSP	Juvenile HOSP	Adult HOSP		
Adult vs. nestling	live birds	4	76	15	84 (0.091)	31.609 (1, 89)	<0.001
	secretions	5	11	22	36 (0.165)	2.809 (1, 13)	0.188
Adult vs. fledgling	live birds	5	71	66	51 (0.193)	0.260 (1, 135)	0.611
	secretions	12	63	60	51 (0.225)	0.020 (1, 126)	0.887

**Table 2:** Rotated component matrix for age effects on semivolatile compounds in house sparrows. All compounds are monoesters. The labeling system uniquely identifies each compound by the total number of carbon atoms it contains and (when applicable) its placement in the repeated pattern shown in Figure 1. Highly significant values (≥0.7) are in bold.

Principal Component				
Compound	1	2	3	4
C24	0.187	0.543	0.053	**0.748**
C24	0.157	0.173	0.25	**0.881**
C24	0.047	0.549	0.279	0.57
C25	0.228	0.54	0.144	**0.773**
C26-1	0.182	0.114	0.454	**0.84**
C26-2	0.237	0.628	0.379	0.295
C26-3	−0.2	0.631	−0.118	0.405
C26-4	0.011	**0.814**	0.278	0.367
C26-5	0.314	0.498	0.238	**0.756**
C27-1	0.236	0.063	0.622	**0.72**
C27-2	0.181	0.669	0.282	0.261
C27-3	−0.103	**0.852**	0.126	0.297
C27-4	0.087	0.657	0.355	0.551
C27-5	0.401	0.489	0.334	0.676
C28-1	0.248	0.068	**0.751**	0.584
C28-2	0.117	**0.793**	0.107	0.35
C28-3	−0.135	**0.836**	0.193	0.245
C28-4	0.206	0.654	0.394	0.477
C28-5	0.478	0.443	0.38	0.62
C29-1	0.19	0.104	**0.869**	0.412
C29-2	0.152	**0.769**	0.143	0.307
C29-3	−0.072	**0.713**	0.3	0.413
C29-4	0.35	0.589	0.379	0.496
C29-5	0.592	0.362	0.405	0.545
C30-1	0.167	0.13	**0.908**	0.306
C30-2	0.365	0.625	0.057	0.338
C30-3	0.088	0.696	0.413	0.288
C30-4	0.511	0.511	0.402	0.412
C30-5	0.667	0.286	0.423	0.471
C31-1	0.186	0.182	**0.913**	0.197
C31-2	0.465	0.467	0.118	0.258
C31-3	0.224	0.632	0.317	0.211
C31-4	0.63	0.371	0.386	0.355
C31-5	**0.727**	0.202	0.439	0.398
C32-1	0.299	0.236	**0.842**	0.202
C32-2	0.481	0.316	0.205	0.156
C32-3	0.144	0.554	0.363	0.133
C32-4	0.681	0.266	0.392	0.33
C32-5	**0.757**	0.135	0.475	0.333
C33-1	0.42	0.314	**0.771**	0.151
C33-2	0.371	0.111	0.118	−0.036
C33-3	0.177	0.441	0.398	0.006
C33-4	**0.754**	0.189	0.392	0.262
C33-5	**0.789**	0.067	0.489	0.285
C34-1	0.532	0.367	0.624	0.205
C34-2	0.216	−0.134	0.058	0.028
C34-3	−0.169	0.273	0.052	0.095
C34-4	**0.747**	0.16	0.44	0.237
C34-5	**0.82**	0.013	0.466	0.253
C35-1	0.634	0.334	0.539	0.201
C35-2	−0.093	0.216	0.002	−0.105
C35-3	**0.85**	0.096	0.262	0.2
C35-4	**0.849**	−0.049	0.406	0.239
C35-5	**0.779**	0.208	0.372	0.191
C36	**0.826**	0.01	0.266	0.175
C36	**0.856**	−0.09	0.336	0.243
C36	**0.778**	0.156	0.206	0.234
C37	0.693	−0.159	−0.019	0.281

## Supplementary Materials

The following are available online at http://www.mdpi.com/2075-4450/9/4/127/s1, Table S1: Live assays only HOSP; Table S2: Secretion assays only HOSP.
